# Noninvasive anatomical assessment for ruling out hemodynamically relevant coronary artery anomalies in adults – A comparison of coronary-CT to invasive coronary angiography: The NARCO study design

**DOI:** 10.1016/j.conctc.2024.101394

**Published:** 2024-11-19

**Authors:** Marius R. Bigler, Anselm W. Stark, Isaac Shiri, Joel Illi, Matthias Siepe, Federico Caobelli, Andreas A. Giannopoulos, Ronny R. Buechel, Andreas Haeberlin, Dominik Obrist, Lorenz Räber, Christoph Gräni

**Affiliations:** a Department of Cardiology, Inselspital, Bern University Hospital, University of Bern, Bern, Switzerland; b Centre for Congenital Heart Disease, Department of Cardiovascular Surgery, Inselspital, Bern, Switzerland; c University Clinic of Nuclear Medicine, Inselspital, Bern University Hospital, Switzerland; d Department of Nuclear Medicine, Cardiac Imaging, University Hospital Zurich, 8091, Zurich, Switzerland; e ARTORG Center for Biomedical Engineering Research, Faculty of Medicine, University of Bern, 3008, Bern, Switzerland

**Keywords:** Anomalous aortic origin of a coronary artery, AAOCA, Dobutamine-volume challenge, FFR, IVUS, SPECT, Assessment of hemodynamic relevance

## Abstract

**Background:**

Anomalous aortic origin of a coronary artery (AAOCA) is a rare congenital heart disease, potentially leading to myocardial ischemia and adverse cardiac events. As the sole presence of AAOCA does not always imply a revascularization, a detailed anatomical and functional analysis is crucial for clinical decision-making. Currently, invasive coronary angiography is the gold-standard method for a thorough hemodynamic assessment of AAOCA. However, due to its invasive nature, the development of noninvasive diagnostic alternatives is desired.

**Methods:**

In the NARCO trial, patients with AAOCA will undergo coronary computed tomography angiography (CCTA) to assess anatomical high-risk features followed by a vessel-based (i.e. invasive measurement with fractional flow reserve and intravascular imaging under a dobutamine-volume challenge) and a myocardium-based (i.e. nuclear imaging) ischemia testing. Comparison of noninvasive and invasive imaging will be performed. Additionally, explorative analysis of post-processing advanced computational fluid dynamics (CFD) and 3D printing will be performed to unravel the pathophysiologic mechanism of myocardial ischemia in AAOCA.

**Aims:**

Our primary aim is to define characteristics of anatomical high-risk features (using CCTA) to rule out noninvasively hemodynamically relevant anomalous vessels in AAOCA patients. The secondary aim is to investigate the underlying pathophysiology of AAOCA-related hemodynamic relevance using advanced techniques such as CFD and 3D printing.

**Conclusions:**

The NARCO trial will help to optimize AAOCA patient selection for revascularization by improving risk stratification and ruling out hemodynamic relevance noninvasively and, therefore, preventing unnecessary downstream testing and/or costly interventions in patients with AAOCA.

## 1 Introduction

Anomalous aortic origin of a coronary artery (AAOCA) is a rare congenital heart anomaly that arises during embryonic development, stemming from abnormal integration of the initially formed subepicardial vascular plexus into the aortic root [1,2]. Conversely to other coronary artery anomalies (such as absent left main stem with two separate ostia for left anterior descending coronary artery and circumflex coronary artery), which do not present any clinical significance [3], AAOCA has been associated with a higher risk for myocardial ischemia and sudden cardiac death (SCD) [4]. Of particular interest are AAOCA with an intramural course (i.e. within the tunica media of the aortic root) [5], as this entity demonstrates a unique pathomechanism of myocardial ischemia with not only fixed stenotic components (similar to atherosclerotic lesions), but often the additional presence of dynamic stenotic components, based on lateral compression of the intramural segment during physical stress [6]. Besides the intramural course, further considered anatomical high-risk features are a slit-like ostium, an acute take-off angle, proximal narrowing of the anomalous segment, as well as a vessel deformation (i.e. an elliptic vessel shape). The sole presence of AAOCA itself or the additional feature of interarterial course (historically called malignant) is not per se an anatomic high-risk feature but rather a surrogate marker for other high-risk features (i.e. intramural course [5]). As the clinical presentation of AAOCA is very heterogeneous and the pathomechanism is complex, clinical decisions need to implement all information, and moving towards revascularization cannot solely rely on symptoms [7]. In fact, while most patients are asymptomatic, encountered symptoms may include many different forms, such as atypical chest discomfort, exercise-unrelated chest pain, dyspnea, dizziness, and palpitations, but as well more typical ischemic symptoms like angina pectoris, exercise-induced dyspnea, syncope, myocardial infarction and rarely SCD [6,[8], [9], [10], [11], [12]]. The latter has been particularly noted in young individuals and was closely associated with intense physical exertion according to autopsy studies [4,10,13]. Further, the current lack of evidence to inform practice guidelines [14,15] leads to ongoing uncertainty in risk assessment and selecting patients for revascularization [16,17]. In parallel, the increasing performance of guideline-conform coronary computed tomography angiography (CCTA) for the evaluation of stable chest pain syndrome [18], especially in the middle-aged and older population for coronary artery diseases assessment, leads to an increase of absolute numbers of coronary artery anomaly detection [5,[19], [20], [21]]. Due to the lack of evidence-based data, combined with the rising numbers of patients detected with AAOCA, the issue of improving clinical management of AAOCA is an urgent unmet need. Hence, we promote a thorough clinical work-up, including the determination of the hemodynamic relevance of the anomaly to make informed treatment decisions [6,22,23]. Invasive coronary angiography combined with intravascular imaging (intravascular ultrasound (IVUS), optical coherence tomography (OCT)) and pressure gradient measurement (fractional flow reserve [24], FFR) during a pharmacologic stress test is the preferred method for a comprehensive assessment, owing to its ability to evaluate the unique pathomechanism of myocardial ischemia in AAOCA. However, its invasive nature with the associated possibility of complications, as well as the uncomfortable rigorous pharmacologic stress test (i.e. dobutamine-volume challenge [6]) poses relevant limitations. Moreover, it represents a “vessel-based” rather than a “myocardium-based” assessment and, thus, neglecting additional physiologic factors contributing to patients’ overall AAOCA risk. Hence, there is a compelling need for a reliable, noninvasive diagnostic evaluation capable of comprehensively assessing the full clinical relevance of AAOCA.

### 1.1 Pathophysiological mechanisms of myocardial ischemia in AAOCA

Guiding the clinical management requires a complete evaluation of the unique pathophysiology of myocardial ischemia in AAOCA. In contrast to the hemodynamic relevance in atherosclerotic coronary artery diseases (CAD), where a fixed reduction of the cross-sectional area causes flow restrictions and, thus, myocardial ischemia, an additional dynamic stenotic component becomes apparent in AAOCA [6].

For the evaluation of the fixed stenotic component, which shares similar pathophysiological characteristics with obstructive CAD, the same assessment tools can be utilized accordingly. Hence, measurement of the cross-sectional area reduction at the ostium (i.e. the slit-like ostium) respectively in the proximal part (i.e. proximal narrowing) in comparison to a distal “reference” diameter allows an evaluation of the fixed component. Additionally, an invasive measurement of the pressure gradient (i.e. FFR) along the anomalous segment, as implemented for the hemodynamic relevance of atherosclerotic lesions, can be applied [25]. Conversely, the dynamic stenotic component becomes only relevant in appearance under stress conditions. Even though the intramural coronary segment experiences a cyclical compression during systole (i.e. “phasic compression”), it usually does not impede diastolic coronary perfusion under normal resting conditions [9,26]. However, vigorous exercise with associated increased cardiac output, heart rate and elevated blood pressure results in an increased aortic wall stress. This, in turn, results in the expansion of the aortic diameter and an intensified lateral compression of the intramural coronary segment during both systole and diastole, thereby impeding diastolic coronary blood flow [6]. Additional exacerbating factors include the shortening of the diastolic phase as well as the aggravation of the acute-take-off angle and the elliptic vessel shape. Consequently, the dynamic stenotic component of hemodynamic relevance in certain AAOCA variants refers to a set of features that specifically emerge during intense physical exercise.

Importantly, the unpredictability of clinical ischemic episodes is influenced by additional factors like the patient's volume status, weight, body composition, heart rate and blood pressure, type of physical activity being performed, affected myocardial mass, and coronary dominance of the anomalous coronary artery, all contributing variably to potential impaired myocardial perfusion [27].

### 1.2 Aims/Hypothesis

Our primary aim is to define characteristics/thresholds of anatomical high-risk features (using CCTA) to rule out noninvasively hemodynamically relevant anomalous vessels in AAOCA patients. An invasively measured FFR (under a dobutamine/volume challenge protocol) will serve as a reference standard to assess hemodynamic relevance. Based on this analysis, we want to implement a noninvasive CCTA-derived high-sensitivity rule-out test for the hemodynamic relevance of AAOCA.

Our primary hypothesis is that FFRdobutamine is associated with the extent and the individual combination of the anatomical high-risk features in patients with AAOCA. Patients will be grouped according to invasive FFRdobutamine (≤ or >0.80). Thus, hemodynamic non-relevant AAOCA with anatomical high-risk features will serve as controls. Assuming the validity of the hypothesis, the study result will allow patients to be deferred from unnecessary heart surgery, downstream testing, and/or sports restrictions.

Our secondary aim is to assess the performance of noninvasive myocardium-based ischemia testing (i.e. nuclear imaging) against vessel-based ischemia testing from invasive stress-evaluation. Further, we want to investigate the underlying pathophysiology of AAOCA-related hemodynamic relevance by using sophisticated, up-to-date computational fluid dynamics (CFD) techniques derived from CCTA and invasive data (in particular IVUS and FFRdobutamine). Analysis will be performed with non-commercially available research software to calculate FFR based on CT images (Siemens cFFR version 3.5.0 or higher Siemens Healthineers) [28], as well as with newly built CFD models with geometries based on CT images and combined CT/IVUS data [29]. All results will be validated against the invasively measured pressure measurements. Further, 3D printing flow-loop analysis will be explored. Last, all patients will undergo clinical follow-up. For the pursuing exploratory analysis, i.e. linking management and outcome to initial anatomical, ischemia testing, postoperative cardiac imaging, CFD and 3D printing models, retrospective as well as prospective data from the University Hospital of Bern registry and other external registries (e.g. University Hospital Zurich) are planned.

## 2 Methods

Consecutive patients will be prospectively recruited from the established specialized clinic for coronary artery anomalies, Department of Cardiology, Inselspital, Bern University Hospital, Bern, Switzerland. All patients will, based on our established internal clinical guideline, undergo a CCTA with quantitative assessment of the anatomical high-risk features. After confirmation of the diagnosis, the patients will be included in the coronary artery anomaly NARCO registry (KEK 2020-00841, Clinical trials NCT04475289). For those with at least one anatomical high-risk feature (i.e. slit-like ostium, acute take-off angle, intramural course, proximal narrowing and/or elliptic vessel shape) an additional comprehensive diagnostic approach with a vessel-based ischemia testing using invasive FFR/IVUS under dobutamine and a myocardium-based ischemia testing (i.e. nuclear imaging, using physical stress or dobutamine stress) will be performed. Exams will be performed within a maximum of 6 months between the assessments. The following explanations will be focused on this subgroup of patients with a potential hemodynamic relevant coronary anomaly. Depending on the routinely acquired results of the invasive FFR and CCTA findings as well as the patients symptoms and individual risk factors (such as competitive sports activity), decisions regarding revascularization versus conservative treatment for the group of patients with a hemodynamically relevant anomaly will be made through an interdisciplinary approach involving interventional cardiologists, cardiovascular imagers, and cardiac surgeons. For the group of patients without evidence of hemodynamic relevance during the stress test and no AAOCA-suspected related persistence of symptoms, no further interventions (e.g. medical therapy and/or sport restriction) will be made. However, all patients will receive a follow-up within the NARCO-registry.

Lastly, CFD with reconstruction of the coronary artery circulation and fluid dynamic calculations will be continuously performed within the study duration without implication on the clinical management. Flowchart 1 graphically demonstrates the planned procedure. For secondary aims such as explorative anatomical, ischemia testing, as well as CFD and 3D printing models linked to management and clinical outcome data, not only prospective-, but also retrospective included patients from the coronary artery anomaly registry will be assessed.

**Fig. 1:**
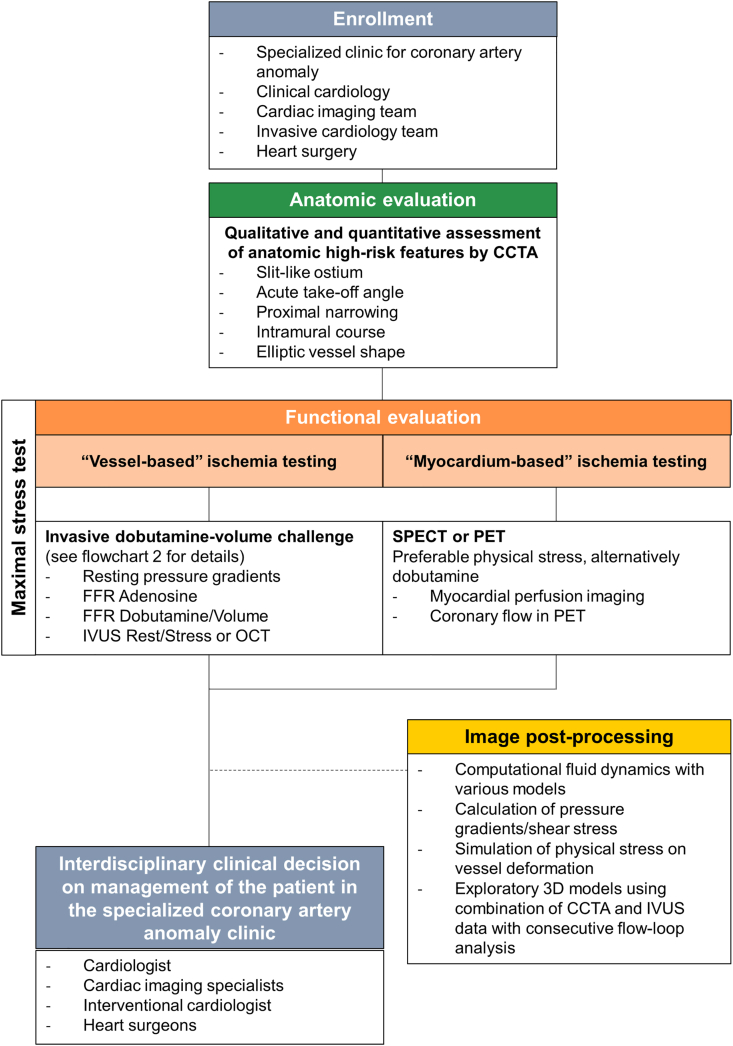
Flowchart 1: Illustration of the study procedure

### 2.1 Anatomical assessment

All subjects will undergo a routinely acquired CCTA imaging. Based on its excellent spatial resolution and advanced post-processing techniques enabling a detailed quantitative assessment of the anatomical high-risk features, CCTA is the preferred imaging modality in the adult population for the anatomical evaluation over magnetic resonance imaging and echocardiography [30]. Of particular interest is the vessel origin, vessel course [31,32] and quantitative assessment of the slit-like ostium (in %) [31], the exact take-off angle (in degrees) [31], the length of the intramural segment (in mm) [31,[33], [34], [35]], the proximal narrowing (also referred as proximal hypoplasia, in %) as well as the extent of vessel deformation (i.e. elliptic vessel shape, dimensionless ratio) [36]. Further, the take-off height (in mm) of the anomalous vessel will be analyzed [37].

### 2.2 Functional assessment

#### 2.2.1 Vessel-based ischemia testing

Patients will undergo left heart catheterization and coronary angiography for diagnostic purposes from the right radial artery approach. Biplane coronary angiography with perpendicular recording of the anomalous segment (required for post-processing analysis) will be performed. In cases with a significant coronary lesion downstream of the anomalous segment, percutaneous treatment will be performed before the study protocol. All patients receive a continuous infusion of saline (max. 3l) after assessment of the resting parameters as preparation for the dobutamine/volume challenge (Flowchart 2).

**Fig. 2:**
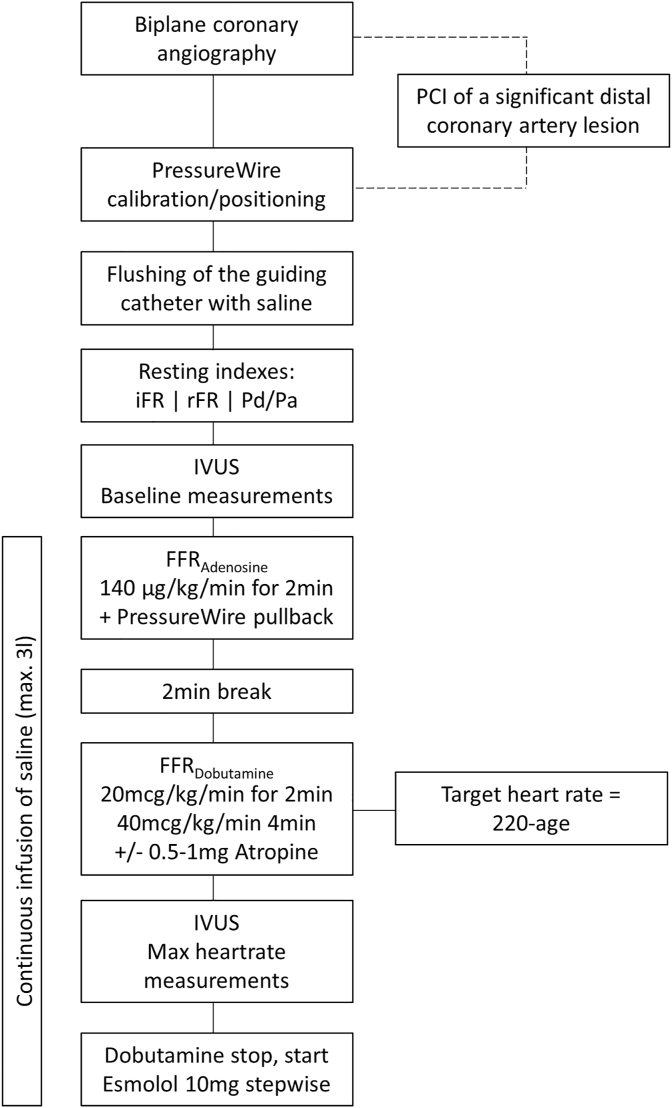
Flowchart 2: Illustration of the study procedure during the vessel-based ischemia testing including the dobutamine/volume challenge

Following diagnostic coronary angiography, a 0.014 inch pressure monitoring angioplasty guidewire (PressureWire™ X Guidewire, Abbott, Chicago, Illinois, United States) will be calibrated and positioned in the distal part of the vessel downstream of the anomalous segment. Next, calculation of resting non-hyperemic pressure ratios (i.e. instantaneous wave-free ratio, resting full cycle ratio and Pd/Pa, all calculated as previously described [38,39]) will be performed. After recording the resting indexes, baseline (i.e. under resting condition) IVUS will be assessed at the site of maximal stenosis (minimal cross-sectional area). IVUS pullback will be conducted at a speed of 0.5 mm/s throughout the suspected lesion. Lesion length, oval-vessel shape and minimal cross-sectional area at rest and during pharmacologic stress will be assessed in the Bern University Hospital Corelab.

Further, FFRadenosine will be assessed during administration of intravenous (i.v.) adenosine (140 μg/kg/min for approximately 2min). FFR will be calculated as the ratio of mean distal (non‐ectopic coronary) pressure to mean aortic pressure and will be considered positive if FFR≤0.80 [40].

Last, dobutamine/volume challenge will be performed by administration of i.v. dobutamine at a rate of 20mcg/kg/min for 2 min, followed by 4 min of i.v. dobutamine at a rate of 40mcg/kg/min. In addition, up to 3 L of saline solution will be administered to counteract cardiac preload reduction and subsequent systemic blood pressure drop by dobutamine. If the maximal heart rate (=220-age) will not be reached after 6 min, 0.5–1 mg of intravenous atropine will be given aiming for the maximum tolerable heart rate. Throughout the stress test, aortic as well as distal coronary artery pressure will be recorded continuously for calculation of FFRdobutamine. At maximal achieved heart rate, the above mentioned IVUS protocol will be repeated for the assessment of the hyperemic values (i.e. evidence of a lateral compression). Afterward, dobutamine will be stopped, and esmolol (10 mg stepwise) will be given to slow down the heart rate.

#### 2.2.2 Myocardium-based ischemia testing

Nuclear cardiac imaging modalities (single-photon emission computed tomography (SPECT) or positron emission tomography (PET)) are widely used for risk stratification and for the assessment of myocardial perfusion in coronary artery disease. Owing to their ability to measure perfusion through the accumulation of radioactive tracers in the myocardium, nuclear cardiac imaging techniques provide a robust evaluation of myocardial perfusion and, thus, an independent evaluation of the hemodynamic relevance of the anomaly. Of note, combination with CT allowing the allocation to the corresponding vessel territory is crucial because of the often altered situation in AAOCA [23,41,42].

In the NARCO-trial, all patients will undergo nuclear imaging if there is no contraindication, using PET or SPECT with physical (i.e. bicycle ergometer) stress test or a pharmacologic stress test (dobutamine + atropine) if possible, striving for a maximal stress burden [43].

### 2.3 Image post-processing

CFD will be performed with dedicated CFD coronary artery software and with general-purpose CFD models [29]. Dedicated CFD models are based on independent, non-commercially available onsite research software developed to estimate FFR from CCTA (cFFR by Siemens Healthineers) [28]. The software allows to calculation of FFR based on the patient's CCTA images after segmentation of the coronary tree. This dedicated CFD model uses fixed assumptions specific to coronary arteries and a simplified model instead of solving the full Navier-Stokes equation, only requiring the user to perform the segmentation of the coronary artery tree. Further analyses will be performed with the use of general-purpose CFD software (e.g. ANSYS 2022R2, Pennsylvania, USA). Lastly, three-dimensional models of the left and right coronary trees will be generated from coronary CTA images using proprietary, purpose-developed software (Mimics, Materialise, Leuven, Belgium) to segment coronary arteries and determine vessel centerlines.

Data from the invasive assessment will be used to validate results of the CFD analysis. The governing Navier–Stokes equations for the conservation of mass and momentum will be solved with the finite volume method (Fluent 14.5, ANSYS). Mean pressure distal to the interrogated lesion will be obtained from the blood flow simulation in the vessel location matching the invasive FFR measurement based on interpretation of the invasive coronary angiography images recording pressure wire position. Matching will be blinded to the invasive FFR measurement. FFRCT values will then be calculated by dividing the mean pressure distal to the coronary anomalous segment (identical to the invasive position) by the mean aortic pressure [44].

Additionally and as an exploratory analysis, we will create 3D models combined of segmented IVUS images and CCTA images [45,46]. These 3D printings will then be used to perform flow-loop analysis [47].

### 2.4 Sample size

Concerning the power analysis, a total sample size of 39 (6 of 39 with abnormal FFR≤0.8) achieves 80 % power (1-beta), with a significance level (alpha) of 0.05 to reject the null hypothesis of an area under the receiver operating characteristic curve less than 0.8 (MedCalc Software Ltd, Ostend, Belgium). The projected allocation ratio (6 of 39) is based on previous literature, that ∼18 % of coronary anomalies are associated with an FFR≤0.80 [[40], [48]]. However, based on rarity of coronary anomalies with associated uncertainites concering the prevalence, we will conservatively include 50 subjects for the primary aim.

## 3 Conclusion

CCTA has emerged as a noninvasive imaging modality, which is widely used to describe anatomic features of AAOCA. Some AAOCA variants are associated with ischemia and adverse cardiac events, whereas most variants may be hemodynamically not relevant and innocent bystanders. It is important to select the optimal patients for further testing and to defer patients who are not at risk for ischemia. However, there is to date no evidence available on how AAOCA patients without risk for ischemia can be ruled out safely based on CCTA-derived anatomy only. The NARCO trial will help to optimize patient selection and risk stratification in order to prevent unnecessary downstream testing and costly interventions in these patients.

## CRediT authorship contribution statement

**Marius R. Bigler:** Writing – original draft, Investigation, Formal analysis. **Anselm W. Stark:** Methodology, Formal analysis, Data curation. **Isaac Shiri:** Writing – review & editing. **Joel Illi:** Writing – review & editing, Software, Methodology. **Matthias Siepe:** Writing – review & editing. **Federico Caobelli:** Writing – review & editing. **Andreas A. Giannopoulos:** Writing – review & editing, Methodology, Conceptualization. **Ronny R. Buechel:** Writing – review & editing. **Andreas Haeberlin:** Writing – review & editing, Methodology. **Dominik Obrist:** Writing – review & editing, Methodology. **Lorenz Räber:** Writing – review & editing, Methodology, Conceptualization. **Christoph Gräni:** Writing – review & editing, Visualization, Validation, Supervision, Software, Resources, Project administration, Methodology, Investigation, Funding acquisition, Formal analysis, Data curation, Conceptualization.

## Data sharing statement

The data that support the study findings will be available upon reasonable request.

## Ethical approval information, institution and number(s)

This study design was approved by the Ethics Committee of the Canton of Bern, Switzerland (KEK 2020-00841) and complies with the Declaration of Helsinki.

## Funding

10.13039/100000001Swiss National Science Foundation Grant Number 200871 Noninvasive anatomical assessment for ruling out hemodynamically relevant coronary artery anomalies - A comparison of coronary-CT to invasive coronary angiography (NARCO) to Christoph Gräni.

The study is further supported by 10.13039/100008273Novartis Foundation for medical-biological Research (application #23B108) and 10.13039/100002129Swiss Heart Foundation (application FF23069).

## Declaration of competing interest

Dr Caobelli has received academic grant support from Siemens Healthineers, Mallinckrodt AG and Tillots AG; and has received speaker honoraria from Siemens Healthineers, Pfizer and Bracco. Dr. Giannopoulos receives grant support from the Promedica Stiftung and the Iten-Kohaut Foundation in collaboration with the USZ Foundation. Dr. Haeberlin has received travel fees/educational grants from Medtronic, Biotronik, Abbott, and Philips/Spectranetics without impact on his personal remuneration. He serves as a proctor for Medtronic. He has received research grants from the Swiss National Science Foundation, the Swiss Innovation agency Innosuisse, the Swiss Heart Foundation, the University of Bern, the University Hospital Bern, the Velux Foundation, the Hasler Foundation, the Swiss Heart Rhythm Foundation, and the Novartis Research Foundation. He is Co-founder and CEO of Act-Inno AG. Dr. Obrist is a member of the advisory board of Novostia.Dr. Räber received research grants to the institution by Abbott Vascular, Biotronik, Boston Scientific, Medis, Sanofi, and Regeneron and consultation/speaker fees by Abbott Vacular, Amgen, AstraZeneca, Canon, Occlutech, and Vifor. Dr. Gräni received funding from the Swiss National Science Foundation, InnoSuisse, Center for Artificial Intelligence in Medicine University Bern, GAMBIT foundation, outside of the submitted work.
